# Medico-economic impact of MSKCC non-sentinel node prediction nomogram for ER-positive HER2-negative breast cancers

**DOI:** 10.1371/journal.pone.0169962

**Published:** 2017-02-27

**Authors:** Hélène Bonsang-Kitzis, Delphine Mouttet-Boizat, Eugénie Guillot, Jean-Guillaume Feron, Virginie Fourchotte, Séverine Alran, Jean-Yves Pierga, Paul Cottu, Florence Lerebours, Denise Stevens, Anne Vincent-Salomon, Brigitte Sigal-Zafrani, François Campana, Roman Rouzier, Fabien Reyal

**Affiliations:** 1 Department of Surgery, Institut Curie, Paris, France; 2 Translational Research Department, RT2Lab Team, Institut Curie, Paris, France; 3 Department of Medical Oncology, Institut Curie, Paris, France; 4 Department of Clinical Epidemiology, Institut Curie, Paris, France; 5 Department of Tumor Biology, Institut Curie, Paris, France; 6 Department of Radiation Oncology, Institut Curie, Paris, France; Western General Hospital, UNITED KINGDOM

## Abstract

**Background:**

Avoiding axillary lymph node dissection (ALND) for invasive breast cancers with isolated tumor cells or micrometastatic sentinel node biopsy (SNB) could decrease morbidity with minimal clinical significance.

**Purpose:**

The aim of this study is to simulate the medico-economic impact of the routine use of the MSKCC non-sentinel node (NSN) prediction nomogram for ER+ HER2- breast cancer patients.

**Methods:**

We studied 1036 ER+ HER2- breast cancer patients with a metastatic SNB. All had a complementary ALND. For each patient, we calculated the probability of the NSN positivity using the MSKCC nomogram. After validation of this nomogram in the population, we described how the patients’ characteristics spread as the threshold value changed. Then, we performed an economic simulation study to estimate the total cost of caring for patients treated according to the MSKCC predictive nomogram results.

**Results:**

A 0.3 threshold discriminate the type of sentinel node (SN) metastases: 98.8% of patients with pN0(i+) and 91.6% of patients with pN1(mic) had a MSKCC score under 0.3 (false negative rate = 6.4%). If we use the 0.3 threshold for economic simulation, 43% of ALND could be avoided, reducing the costs of caring by 1 051 980 EUROS among the 1036 patients.

**Conclusion:**

We demonstrated the cost-effectiveness of using the MSKCC NSN prediction nomogram by avoiding ALND for the pN0(i+) or pN1(mic) ER+ HER2- breast cancer patients with a MSKCC score of less than or equal to 0.3.

## Introduction

Sentinel node (SN) biopsy is a standard procedure in early-stage invasive breast cancer [1,2]. Completing axillary lymph node dissection (ALND) remains the standard care for patients with disease-positive SN in many countries. However, 40 to 70% of patients with a metastatic sentinel lymph node biopsy turn out to have no additional disease-positive nodes [3–6]. Axillary management has changed recently, with a trend toward diminishing the use of ALND especially in patients with isolated tumor cells or micrometastatic SN whose prognostic value remains controversial [7–16]. Even for patients with 1–2 macrometastatic sentinel nodes when associated with conserving surgery and radiotherapy, ALND was abandonned in US (ACOZOG Z11).

Histopathologic parameters are helpful to predict additional non-sentinel lymph node metastases among patients with a positive SN biopsy: tumor size, tumor grade, lymphovascular invasion, maximal diameter of the sentinel node metastasis, number of positive and negative SN biopsies, extracapsular extension [3–5,11,17–22].

Several nomograms have been developed to predict the risk of non-sentinel node metastases in patients with a tumor-positive sentinel node [5,18,19].

The MSKCC nomogram and the Tenon score are the more accurate predictive models [23,24]. Moreover, the MSKCC (Memorial Sloan Kettering Cancer Center) nomogram has been validated in patients with estrogen-receptor positive HER2-negative subgroup [25] and patients with micrometastatic SN [26].

The aim of this study was to see if we had robust determinent to help us define the rate of non-sentinel-node metastases in ER-positive HER2-negative breast cancer patients with a metastatic SN, using the MSKCC nomogram. We therefore evaluated the medico-economic impact of the identified threshold.

### Ethical approval

All experiments were performed retrospectively and in accordance with the French Bioethics Law 2004–800, the French National Institute of Cancer (INCa) Ethics Charter and after approval by the Institut Curie review board and ethics committee (Comité de Pilotage of the Groupe Sein). In the French legal context, our institutional review board waived the need for written informed consent from the participants. Moreover, women were informed of the research use of their tissues and did not declare any opposition for such research. Data were analyzed anonymously.

## Patients and methods

### Patients

We retrospectively studied 1036 patients treated at Curie Institute for operable breast cancer between 2006 and 2012. Each one had estrogen-receptor positive HER2-negative tumors with a metastatic sentinel lymph node (macrometastatic pN1, micrometastatic pN1mic, or isolated tumor cells pN0i+). They all also had a complementary ALND performed at level I and II of Berg floor.

Sentinel node (SN) detection was performed using radioisotope alone or combined with the colorimetric method. Technetium 99mTc (100 MBq) was injected in the external periareolar area the day before surgery. Lymphoscintigraphic imaging was not always performed. When no axillar radioactivity was detected, 2ml of blue patent was injected into the periareolar area 10 minutes before surgery. Routine intraoperative examination of sentinel nodes was complimentary performed.

All patients had a complementary axillary lymph node dissection (ALND) during the same procedure if the SN was found metastatic by frozen section, or if after the post-operative histopathologic analysis.

The following histopathologic criteria were retrospectively obtained from the anatomopathologic reports: tumor type; clinical tumor size according to the pTNM classification; tumor grade according to the Ellston and Ellis grading system; lymphovascular invasion or not; type of metastatic SN; maximal diameter of the SN metastases; number of positive SN and non-SN biopsies.

We calculated the probability of the non-SN positivity for each patient using the MSKCC (Memorial Sloan Kettering Cancer Center) nomogram after surgery, using the website: http://www.mskcc.org/mskcc/htlm/5794.cfm. This calculator is based on the following criteria: pathological size, tumor type and grade, number of positive SN, SN detection method, number of negative SN, lymphovascular invasion, multifocality, ER positivity.

### Statistical analysis

First we performed a descriptive analysis of our population using the following criteria: patient’s age, tumor size, grade and type, lymphovascular invasion or not, type of SN metastases, total number of SN and non-SN involvement.

Secondly, to demonstrate the exportability of the MSKCC prediction nomogram to our population, we validated this nomogram using several criteria: discrimination, calibration, false negative rate, sensitivity, specificity, negative-predicting value, positive-predicting value, models’ efficacy.

The discrimination power was evaluated by the area under the ROC (Receiver Operating Characteristic) curve (AUC). AUC indicates the probability that the MSKCC score place a positive patient in front of a negative patient. In practice, to build the ROC curve, we calculated the MSKCC score for each patient. Scores were then ranked. The ROC curve plot for each value of the MSKCC score (between 0 to 1), the false positive rate (1-specificity) according to sensitivity.

The calibration of the test was assessed graphically, by the calibration plot curve, obtained by linear regression. We tested the likelihood ratio of the zero hypothesis alpha = 0 and beta = 1 using a Chi square test on two degrees of freedom.

The clinical interest of this model was based on the analysis of the false negative rate, the sensitivity, specificity, positive and negative-predictive values.

**Fig 1:**
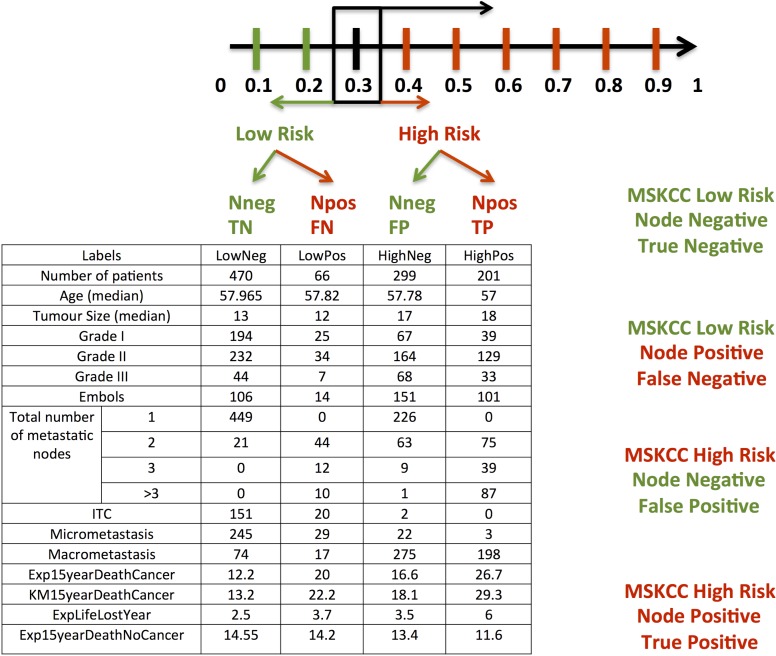
Patient characteristics in every subgroup depending on the threshold score value between 0.1 and 0.9. (True Negative (TN), False Negative (FN), True Positive (TP), False Positive (FP)). Example given for a threshold value = 0.3. *ITC = Isolated Tumor Cells*.

We defined a score threshold of the MSKCC nomogram after a sensitivity analysis to determine the score that gives the optimal sensitivity and specificity for predicting the absence of non-sentinel lymph node macrometastasis. This cutoff score was chosen to divide our population in two groups of patients of great clinical interest according to the type of NSN metastases: low-risk patients below the threshold score (mostly micrometastases or isolated tumor cells), and high-risk patients above the threshold score (mostly macrometastases). Furthermore, this threshold was selected to have a predicted probability of macrometastasis less than 10% and a false negative rate less than or equal to 10%. Fig 1 illustrates how the patients’ characteristics spread as the threshold value changed from 0.1 to 0.9.

Finally, we performed a simple economic simulation study to estimate the total cost of caring for patients treated according to the MSKCC predictive nomogram results. The model used is a decision-making tree.

This type of model represents the possible alternatives for an individual as per her tree, following a timeline. The “roots” of the tree represent the initial decision and its alternatives. Each branch represents a possible sequence; associated to each knot/each event, is its average cost. Once the structure of the tree has been determined, and the probabilities identified we can measure and calculate the final cost for each branch. That is, the calculation of the different possibilities of distribution in our population according to the limits that vary from 0.1 to 0.9.

**Fig 2:**
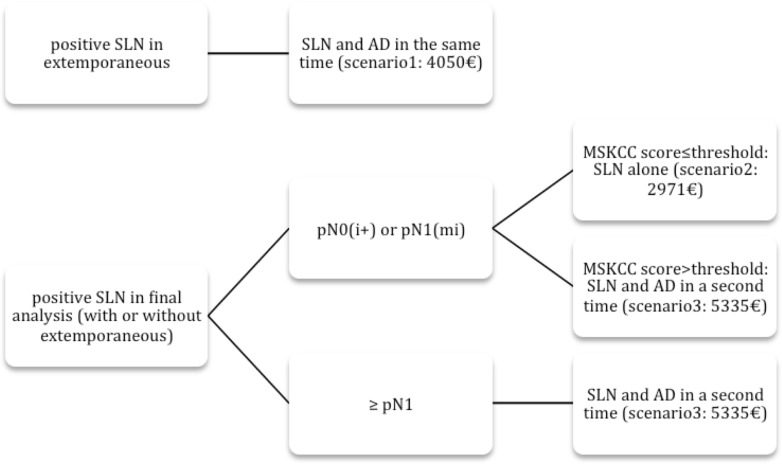
Decision-making tree according to the Sentinel Lymph Node Biopsy results and the MSKCC score. SLN = Sentinel Lymph Node. AD = Axillary Dissection.

Fig 2 illustrates for each patient the three possible different scenari (and their associated cost), depending on whether the SN extemporaneous exam is performed or not, on the type of SN metastases and the non SN prediction nomogram. Costs of the SN procedure were obtained from the medico-economic evaluation as compared to the axillary lymph node dissection in operable breast cancers (Rapport 2008-STIC 2004, INCA-DHOS). Procedural costs: 2971€ for the SN procedure only, 4050€ for the SN biopsy and ALND at the same time, 5335€ for a SN biopsy and a delayed ALND. These costs included the SN extemporaneous exam. We substracted its cost, an average of 54€ per procedure, in the procedures without extemporaneous exam. All costs were calculated in Euros.

### First, when we used a SN extemporaneous exam strategy

The number of intraoperative positive SN leading to an immediate ALND was estimated based on the results of a retrospective study (B.Sigal) evaluating all the intraoperative SN procedures results between 2007 and 2008 in the Curie Institute. In this study, false negative rates of the extemporaneous exam were reported varying from 10% for pN1 tumors, 89% for pN1(mic) tumors and 100% for pN0(i+) tumors (S1 Table). So, for each MSKCC score value, we consider that 90% of pN, 11% of pN1(mic) and 0% of pN0(i+) tumors underwent ALND in the same procedure (4050 euros by patient), 10% of pN, 89% of pN1(mic) and 100% of pN0(i+) tumors were not detected as positive in extemporaneous exam so underwent SN alone (2971 euros by patient) or ALND in a second procedure (5335 euros by patient) according to the final pathological SN status and the MSKCC score value over the threshold.

### Second, when we used a non SN extemporaneous exam strategy

For each MSKCC score value, patients underwent SN alone (2917 euros by patient) or ALND in a second procedure (5281 euros by patient) according to the final pathological SN status and the MSKCC score value over the threshold.

Analyses were performed using R statistical analysis software (http://cran.rproject.org).

## Results

### Population

1036 patients had a breast surgery for early stage invasive ER-positive HER2-negative tumors with metastatic SN biopsy between 2006 and 2012 in the Curie Institute. Their mean age was 57.7 years. There were 86% ductal invasive and 14% lobular invasive carcinomas. There were 54% grade 2 vs 31% grade 1 and 14.6% grade 3 tumors. Tumors were classified as pT1 in 80% of cases (vs 18% of pT2 and less than 1% of pT3). There was lymphovascular invasion in 36% cases. All patients had a metastatic SN biopsy. 54% had a macrometastasis (>2mm), 29% had a micro-metastasis (<2mm) and 17% had isolated tumor cells in the SN samples. There was only one positive SN found in 85% of patients. There was no metastatic non-SN biopsy in 74% of patients. pNode classification changed from 1–3 to more than 3 nodes in 93 cases (9%) after complementary ALND (Table 1).

**Table 1:** Population baseline characteristics.

1036 ER positive HER2 negative tumor-positive sentinel node breast cancer		
Clinical and Pathological Characteristics		N (%)—Median (range)
**Age at diagnosis**	**Median (range)**	57.7 (31–85)
	**30–50**	273 (26%)
	**50–60**	352 (34%)
	**60–90**	411 (40%)
**Tumor Type**	**Ductal**	894 (86%)
	**Lobular**	142 (14%)
**Tumor size (mm)**	**Median (range)**	15 (0–100)
	**pT1**	834 (80%)
	**pT2**	192 (18%)
	**pT3**	0 (0.9%)
**LVI**	**Positive**	372 (36%)
**Elston-Ellis Grade**	**I**	325 (31%)
	**II**	559 (54%)
	**III**	152 (14.6%)
**Detection of metastatic sentinel nodes**	**ITC**	173 (17%)
	**Micrometastasis**	299 (29%)
	**Macrometastasis**	564 (54%)
**Number of metastatic sentinel nodes**	**1**	882 (85%)
	**2**	128 (12%)
	**3**	21 (2%)
	**>3**	5 (0.5%)
**Total number of metastatic nodes**	**0**	769 (74%)
	**1**	139 (13%)
	**2**	50 (5%)
	**3**	28 (3%)
	**>3**	5 (5%)
**pN change from 1–3 to more than 3 nodes**		93 (9%)

### Validation of the MSKCC non-sentinel node prediction nomogram in our population

**Fig 3:**
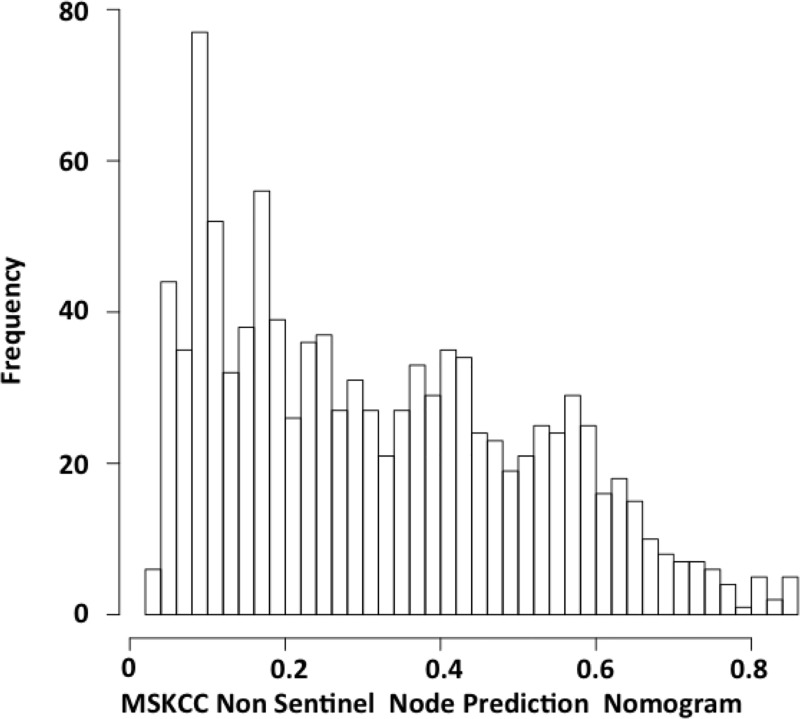
MSKCC Non-Sentinel Node Prediction nomogram scores distribution in our population.

The median MSKCC predicted probability score was 0.3 in our population. 932 patients (90%) had a predicted probability score lower than 0.6 (Fig 3).

**Fig 4:**
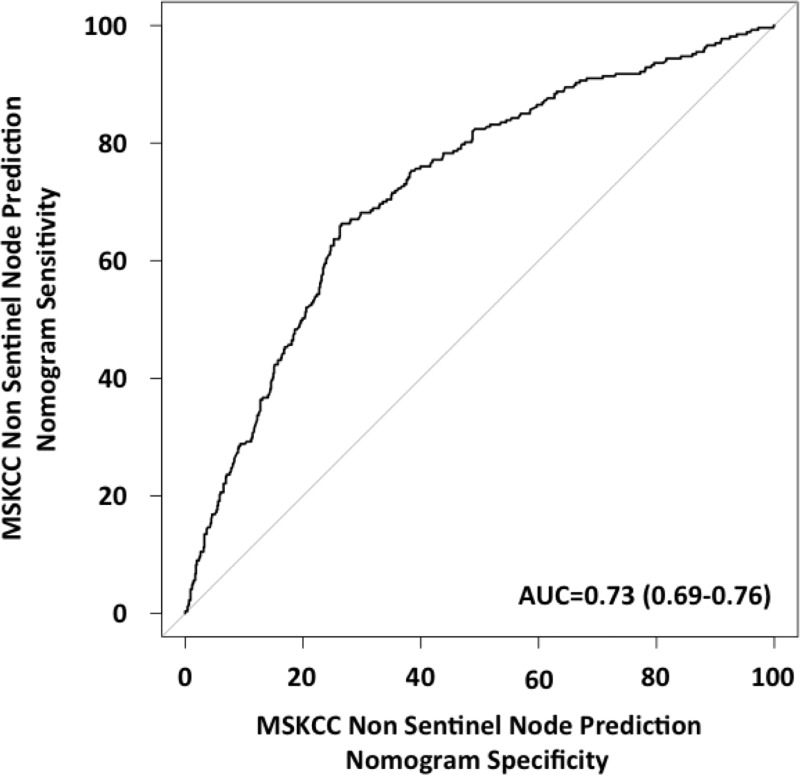
ROC curve for the Non-Sentinel Node Prediction Nomogram (MSKCC) in our population.

The MSKCC Non-Sentinel Node Prediction nomogram in our population was discriminating with an area under the ROC curve of 0.73 IC95% = [0.69–0.76] (Fig 4). Its calibration accuracy was satisfactory with non-statistically different predicted and observed probabilities.

### Patient distribution according to a MSKCC non-sentinel node prediction nomogram threshold set at 0.3

Afterwards, we varied the MSKCC nomogram threshold from 0.1 to 0.9 and observed how the population spread in each case. The threshold of 0.3 was of great interest because it was equivalent to the median score obtained in our population, and also seemed to discriminate accurately the type of SN metastases.

**Fig 5:**
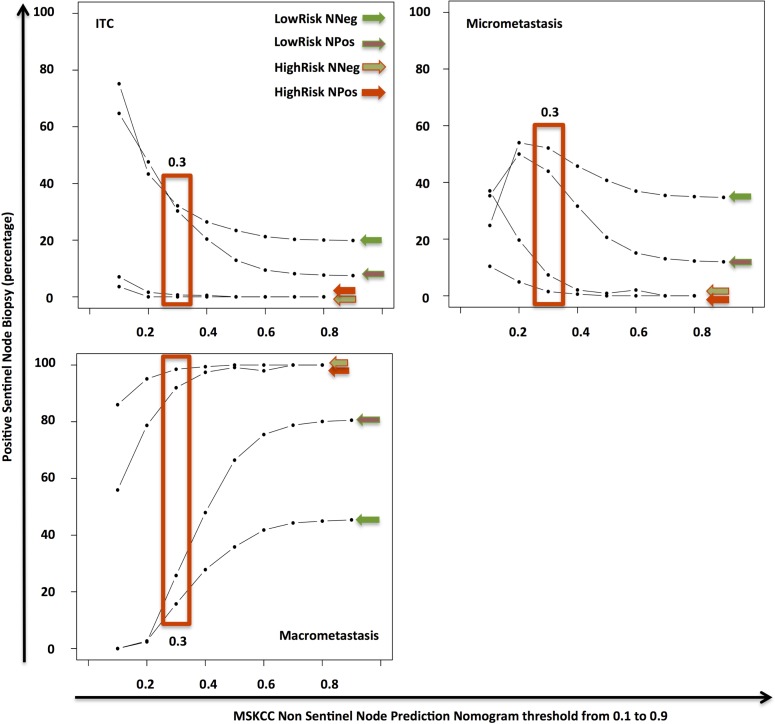
Type of SN metastases according to the MSKCC Non-Sentinel Node Prediction nomogram score in True Negative (TN), False Negative (FN), True Positive (TP), False Positive (FP) patients. True Negative = LowRisk NNeg, False Negative = LowRisk NPos, True Positive = HighRisk NPos, False Positive = HighRisk NNeg.

**Fig 6:**
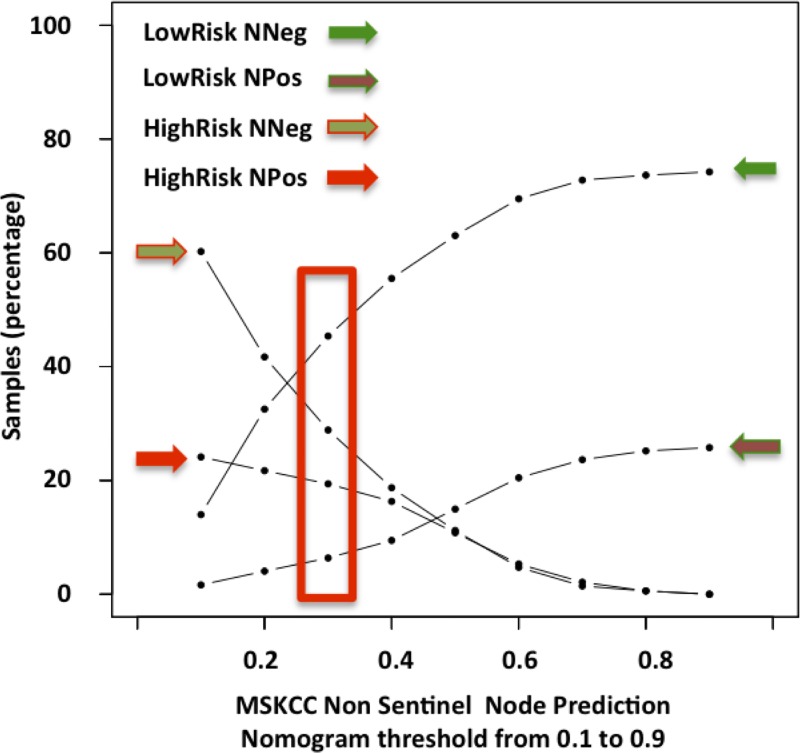
True Negative (TN), False Negative (FN), True Positive (TP), False Positive (FP) rates according to the Non-Sentinel Node Prediction Nomogram score (MSKCC).

Indeed, we found mainly small volume metastases below this threshold: 94.5% of micrometastases or isolated tumor cells versus 5.7% of macrometastases (Fig 5). Moreover, the score threshold of 0.3 accurately predicted 64.8% of the patients (671/1036 patients) and provided a false negative rate of 6.4% (66/1036 patients) and a false positive rate of 28.9% (299/1036 patients) (Fig 6). This corresponded to a sensitivity of 75.3%, a specificity of 61.1% and a predictive accuracy of 65%.

### Patient characteristics with a threshold score set at 0.3 (Table 2)

The median age of patients with a MSKCC score less than or equal to 0.3 was not significantly different from the patients with a MSKCC score greater than 0.3 (58 years vs 57 years, p = 0.5).

**Table 2:** Population’s distribution with a nomogram threshold set at 0.3 (MSKCC).

1036 ER positive HER2 negative tumor-positive sentinel node breast cancer. Threshold = 0.3 (MSKCC)				
Patients status (MSKCC score)		Low Risk (≤ 0.3) 536 patients	High Risk (> 0.3) 500 patients	p value
**Clinical and Pathological Characteristics**		**N (%)—Median (range)**	**N (%)—Median (range)**	
**Age at diagnosis**	**Median (range)**	58 (31–82)	57 (31–85)	
	**30–50**	133 (24.8)	140 (28.0)	0.5
	**50–60**	186 (34.7)	166 (33.2)	
	**60–90**	217 (40.5)	194 (38.8)	
**Tumor Type**	**Ductal**	468 (87.3)	426 (85.2)	0.37
	**Lobular**	68 (12.7)	74 (14.8)	
**Tumor size (mm)**	**Median (range)**	12 (0–60)	17 (2–100)	
	**pT1**	484 (90.5)	350 (70.6)	**5.4 10**^**−16**^
	**pT2**	50 (9.3)	142 (28.6)	
	**pT3**	1 (0.2)	4(0.8)	
**LVI**	**Positive**	120 (22.4)	252 (50.4)	**< 2.2 10**^**−16**^
**Elston-Ellis Grade**	**I**	219 (40.6)	106 (21.2)	**7.4 10**^**−13**^
	**II**	266 (49.6)	293 (58.6)	
	**III**	51 (9.5)	101 (20.2)	
**Detection of metastatic sentinel nodes**	**ITC**	171 (31.9)	2 (0.4)	**< 2.2 10**^**−16**^
	**Micrometastasis**	274 (51.1)	25 (5.0)	
	**Macrometastasis**	91 (17.0)	473 (94.6)	
**Number of metastatic sentinel nodes**	**1**	513 (95.7)	369 (73.8)	**< 2.2 10**^**−16**^
	**2**	23 (4.3)	105 (21.0)	
	**3**	0	21 (4.2)	
	**>3**	0	5 (1.0)	
**Total number of metastatic nodes**	**0**	470 (87.7)	299 (59.8)	**< 2.2 10**^**−16**^
	**1**	45 (8.4)	94 (18.8)	
	**2**	11 (2.0)	39 (7.8)	
	**3**	6 (1.1)	22 (4.4)	
	**>3**	4 (0.7)	46 (9.2)	

### Tumor characteristics: Type, size, grade, lymphovascular invasion (LVI)

The proportion of ductal and lobular invasive carcinomas was not significantly different between the two sub-groups (p = 0.37).

Patients with a MSKCC score greater than 0.3 had a significantly larger tumor size (mean size 17mm vs 12mm, 71% of pT1 and 29% of pT2 vs 90% and 9% respectively, p = 5,4.10^−16^), a significantly higher grade (21% of grade 1, 59% of grade 2 and 20% of grade 3 vs 41%, 50% and 9% respectively, p = 7,4.10^−13^), with LVI in 50% of cases (vs 22%, p = 2,2.10^−16^).

### Characteristics of the metastasis in the SN samples: Type of metastasis, total number of metastatic nodes

A MSKCC score lower than 0.3 corresponded mainly to isolated tumor cells or micrometastasis (<2mm) found in the SN biopsies. Conversely, a score greater than or equal to 0.3 corresponded to macrometastasic SN (32% of pN0(i+), 51%>pN1(mic) and 17% of pN1 vs 0.4%, 5% and 95% respectively, p = 2,2.10^−16^).

Patients with a MSKCC score lower or equal to 0.3 had a significantly lower number of metastatic SN (96% had only one metastatic SN, 4% had twice, none more than two vs 74%, 21% and 5% respectively, p = 5,4.10^−16^), with a non-metastatic complementary ALND (88% vs 60%, p = 2,2.10^−16^).

### «False negative» patient characteristics

In our population database, we observed 66 « false negative » cases (6.3%) with a threshold of 0.3 (MSKCC). Among these 66 patients, if we considered only patients aged under 70 years (52 patients out of 1036; 5%) without macrometastatic SN (39 patients out of 1036; 3.7%), 38 patients out of 39 had less than 3 metastatic lymph nodes after ALND. Which makes only one patient a real «false negative» case, changing from pN1 status to pN2 status (1 out of 1036; 0 .1%).

Patients’ mean age was 57.8 years and 19% were older than 70 years. Tumors presented the following histopathologic criteria: mean size of 12mm, 37.9% of tumor grade 1, 51.5% of grade 2, 10.6% of grade 3, 21.2% of lymphovascular invasion. The final SN analysis reported 30.3% of pN0(i+), 43.9% of pN1(mic), 25.8% >pN1, total number of metastatic SN was two in 66.6% cases, three or more in 33.3% cases. The estimated breast cancer related death at 15 years was 22.2% (www.lifemath.net) (Figs 5 and 6).

### Economic simulation study for a threshold set at 0.3

The economic simulation study was done according to two different scenarios whether the SN extemporaneous exam was performed or not. Each case could correspond to three situations, depending on the type of SN metastases and the non-SN prediction nomogram. Fig 2 illustrates the decision-making tree. The number of intraoperative positive SN leading to an immediate ALND was estimated based on the results of a retrospective study (B.Sigal, S1 Table). The study evaluated all the intraoperative SN procedures results between 2007 and 2008 in the Curie Institute. In this study, false negative rates of the extemporaneous exam were reported varying from 10% for pN1 tumors, 89% for pN1(mic) tumors and 100% for pN0(i+) tumors.

Then we calculated the population’s total cost of caring for every score value between 0.1 and 0.9 (according to the decision-making tree in Fig 2). A score threshold set at 0.3 could reduce the total cost by 1 051 980 euros without SN extemporaneous exams, and by 979 775 euros with SN extemporaneous exams.

**Fig 7:**
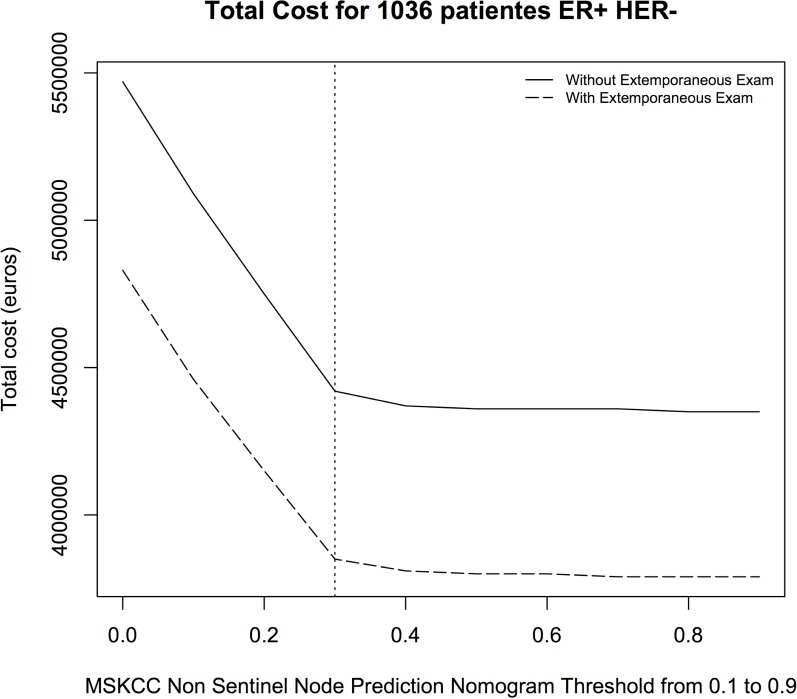
Total cost depending on the threshold set for the MSKCC Non-Sentinel Node Prediction Nomogram with or without Sentinel Node extemporaneous exam (red and blue curve respectively).

If we vary the threshold towards the 0.9, the reduction of total costs appears to be minimal (63 828 euros maximum without SN extemporaneous exams and 59 100 euros maximum with SN extemporaneous exams), the total number of false negative scores increasing substantially (red curve: with SN extemporaneous exam, Blue curve: without SN extemporaneous exam. Fig 7).

Moreover, the SN extemporaneous exam reduces the total cost by 639 241 euros (due to the completion ALND performed during the same procedure).

Finally, the SN extemporaneous exam procedure reduces the total cost by 1 619 016 euros, compared to a procedure without SN extemporaneous exam with a secondary ALND performed for all metastatic SN after postoperative analysis.

## Discussion

Sentinel lymph node biopsy is now a standard of care for axillary staging in early-stage invasive breast cancer (clinically node negative, tumor size ≤ 30mm commonly) [27,28], thus sparing patients with tumor-free SN biopsy from axillary lymph node dissection. This minimally invasive procedure is associated with less morbidity than ALND and gives accurate nodal-staging information.

This ultrastaging increases SN metastases detection. Occult micrometastases in SN classified as negative on initial evaluation can be positive on more comprehensive histologic evaluation.

Completion ALND remains standard treatment for patients with axillary metastases identified on SN biopsy in many countries.

Routine axillary dissection has been largely abandoned in US for patients who fit the criteria T1/T2 tumor, ER/PR+, less than 3 positive sentinel nodes with breast conserving surgery and whole breast radiotherapy, accordingly to ACOZOG Z-0011 clinical trial [29]. But french guidelines don’t recommend the same practice yet. For example, at Curie Institute, our guidelines of treatment did not recommend ALND for isolated tumor cells. We realized ALND for micrometastasis for patients < 40 years or tumors ≥ T2 without other indication of chemotherapy and systematically for macrometastasis.

It remains unclear whether isolated tumor cells or micrometastases represent an adverse prognostic indicator and whether ALND should be carried out in such cases [7–16]. Recent studies report minimal medical gain/impact in terms of locoregional recurrence and progression free survival in patients with SN metastasis pN0(i+) or pN1(mic) without completion ALND [30]. The « American College of Surgeons Oncology Group » studied the prognostic value of micrometastatic SN biopsies (ACOSOG-Z0010 et Z0011 trials) [29,31]: 5000 patients (clinically T1-2, node negative) with a metastatic SN treated by breast lumpectomy and radiotherapy, were then randomly assigned to a completion ALND or no further axillary treatment. This study showed no survival gain after completion ALND, as compared to the SN procedure only in patients with one or two positive SN (median follow-up of 6.3 years).

Results of the retrospective study by Galimberti et al. [32] concerning 377 patients with pN0(i+) or pN1(mic) SN without axillary dissection nor axillary radiotherapy are concordant with the Z0011 trial. Axillary recurrence at 5 years was seen in less than 2.4% of the patients and progression free survival was more than 97.3%. The « International Breast Cancer Study Group » trial (IBCSG 23.01) randomly assigned patients with micrometastatic SN in two subgroups: completion ALND or no further axillary treatment [33]. These results argued for sparing many patients an early breast-cancer axillary dissection, especially when the sentinel node is minimally involved. This reduced surgical complications related to axillary dissection with no adverse effect on survival. However, data from the Z0011 trial remain controversial because of the study’s lack of statistics (insufficient number of patients) and a poor external validation. Plus, 60% of the patients with metastatic SN did not meet the Z0011 inclusion criteria. Guth et al. [34] tested the impact of Z0011 trial on an European population. The application of the Z0011 trial led to the omission of completion ALND in less than 10% of all SN procedures, which was not optimal for changing clinical practice.

In a population-based observational study in the USA, Wasif et al [35] showed the underuse of ALND for the management of SN micrometastases: 40% of 5000 patients with SN micrometastases had no further nodal surgery. These data lead to the aim of our study. We accurately demonstrate that a completion ALND may be omitted in the management of patients with micrometastatic SN or isolated tumor cells.

Patient distribution study according to the MSKCC Non-Sentinel Node Prediction nomogram allowed us to set a score threshold. Below this threshold, the use of a completion ALND may reasonably be omitted.

We demonstrated that the threshold of 0.3 divided the patients into two subgroups (low- and high-risk of having involved non-SN), and that it discriminated the type of SN metastases. Below this threshold, the majority of SN metastases corresponded to micrometastases or isolated tumor cells (98.8% of patients with ITC, 91.6% of patients with micrometastases had a MSKCC score under 0.3). False negative rate of prediction was 6.4% with this threshold (the FN cases included more often grade 2 or 3 tumors or a macrometastatic SN than the « true negative » cases).

In our economic simulation study, only patients with macrometastases, or pN1(mic) and pN0(i+) SN with a MSKCC score above 0.3 did undergo a completion ALND. Indeed, patients with nodal micrometastases are a heterogeneous population and some subsets have a higher risk of axillary recurrence as studied in a large population-based series [36]. Thus, with this management of the axillar nodes, 43% of ALND could be avoided in our study, reducing the costs of caring by 1 051 980 Euros among the 1036 patients with involved SN. Above this threshold, the false negative rate was too high and the cost-effectiveness of the test lower.

Intraoperative assessment of sentinel nodes allows immediate axillary dissection when metastasis is found in the SN [37]. Two procedures of intraoperative SN assessment (imprint cytology or frozen-section analysis) have similar sensitivity and specificity, with false negative rates ranging from 5 to 70% in the literature [38]. False negative rates obtained by intraoperative exam range from 10% for pN1 tumors, 89% for pN1(mic) to 100% for pN0(i+) in the Curie Institute (S1 Table). Thus, in our study, 90% of the patients with SN macrometastases are readily identified by the extemporaneous SN exam and can benefit from an immediate ALND. The extemporaneous exam reduced the costs of caring by 639 241 euros, by allowing an immediate completion ALND for approximately 95% of the patients concerned.

A delayed completion ALND should concern only 10% of the patients with macrometastates left (5.4% of the patients with a positive SN), which reduces the costs of caring by additionnal 979 775 euros.

Intraoperative SN assessment allows patients with metastatic SN to have an immediate complete axillary dissection which is a cost-effective procedure.

## Conclusion

Our study demonstrated the cost-effectiveness of using the MSKCC Non-Sentinel Node Prediction nomogram by sparing from the completion ALND the pN0(i+) or pN1(mic) patients with a MSKCC score less than or equal to 0.3.

Indeed, omitting the completion axillary dissection in the case of micrometastases or isolated tumor cells found on SN biopsy in early breast cancer could be of great interest (reducing the ALND’s morbidity and health care costs). On the basis of the available literature, there is minimal clinical significance in terms of axillary progression or recurrence-free survival in the absence of complementary ALND in these cases. Moreover, axillar surgery as a locoregional treatment starts to be controversial given the contribution of adjuvant treatments in breast cancer.

This strategy could be used for selected patients with estrogen-receptor positive HER2-negative tumors less than 20mm, pN1(mic) or pN0(i+), which correspond to a MSKCC score ≤ 0.3.

Only 57% patients should undergo a complementary ALND in our study, which would reduce the cost of care by 1 051 980 euros.

90% of the patients with identified macrometastases on SN biopsy can benefit from an immediate ALND due to the intra-operative exam of SN.

Intraoperative assessment of SN allows immediate axillary dissection when metastases are found in the SN. In the 90% of patients with positive nodes, disease will be detected intraoperatively. Only 10% of the patients with macrometastases would undergo a delayed ALND, which correspond to 5.4% of our patients with a positive SN biopsy. This standard of care procedure would reduce the total health costs by additionnal 567 036 euros.

## Supporting information

S1 Table Nodal status determination by frozen section examination compared to definitive nodal status. (XLSX)
